# Human Empathy, Personality and Experience Affect the Emotion Ratings of Dog and Human Facial Expressions

**DOI:** 10.1371/journal.pone.0170730

**Published:** 2017-01-23

**Authors:** Miiamaaria V. Kujala, Sanni Somppi, Markus Jokela, Outi Vainio, Lauri Parkkonen

**Affiliations:** 1 Department of Equine and Small Animal Medicine, Faculty of Veterinary Medicine, PL, University of Helsinki, Helsinki, Finland; 2 Department of Neuroscience and Biomedical Engineering, Aalto University School of Science, Aalto, Espoo, Finland; 3 Psychology, Institute of Behavioural Sciences, Faculty of Behavioural Sciences, University of Helsinki, Helsinki, Finland; Universita degli Studi di Pisa, ITALY

## Abstract

Facial expressions are important for humans in communicating emotions to the conspecifics and enhancing interpersonal understanding. Many muscles producing facial expressions in humans are also found in domestic dogs, but little is known about how humans perceive dog facial expressions, and which psychological factors influence people’s perceptions. Here, we asked 34 observers to rate the valence, arousal, and the six basic emotions (happiness, sadness, surprise, disgust, fear, and anger/aggressiveness) from images of human and dog faces with Pleasant, Neutral and Threatening expressions. We investigated how the subjects’ personality (the Big Five Inventory), empathy (Interpersonal Reactivity Index) and experience of dog behavior affect the ratings of dog and human faces. Ratings of both species followed similar general patterns: human subjects classified dog facial expressions from pleasant to threatening very similarly to human facial expressions. Subjects with higher emotional empathy evaluated Threatening faces of both species as more negative in valence and higher in anger/aggressiveness. More empathetic subjects also rated the happiness of Pleasant humans but not dogs higher, and they were quicker in their valence judgments of Pleasant human, Threatening human and Threatening dog faces. Experience with dogs correlated positively with ratings of Pleasant and Neutral dog faces. Personality also had a minor effect on the ratings of Pleasant and Neutral faces in both species. The results imply that humans perceive human and dog facial expression in a similar manner, and the perception of both species is influenced by psychological factors of the evaluators. Especially empathy affects both the speed and intensity of rating dogs’ emotional facial expressions.

## Introduction

Facial expressions play an important part in nonverbal social communication among us humans (for reviews, see e.g. [1,2]) as among other mammals (for reviews, see [3,4]). Human sensitivity for others’ facial expressions facilitates better comprehension of the emotions, moods, attitudes and aims of the conspecifics. Likewise, humans also pay attention to the emotional expressions of non-conspecifics. Domestic dogs (*Canis familiaris*) are of special interest in this context, since they have underwent a long domestication with humans [5,6] and have excellent social communication skills (e.g. [7]). Furthermore, dogs have many of the same muscles that produce facial expressions in humans [8], and their facial expressions are connected to the affective situation [9]. Dogs can differentiate emotions displayed by either human or dog facial expressions [10–12], and they also show rapid facial mimicry in response to a conspecific expression during play [13]. Facial expressions thus provide important information of conspecific emotions also for dogs; they reflect at least some of their emotional states by the face and react to others’ expressions accordingly.

Generally, humans agree that animals such as dogs experience emotions [14–16]—especially basic emotions stated as evolutionary adaptive [17–20]. Human observers show high consistency in describing animal affective behavior [21,22], in classifying dogs’ emotional behavior seen from a video [23,24] and evaluating dogs’ barks in emotional situations [25]. Overtly friendly behavior of a dog is the most easily recognized by humans, whereas dog’s aggression or fear appears more difficult to detect [23,24,26]. Regarding canine facial expressions, valence is discriminated by human observers as accurately as expressions of human children [9]. However, dog facial expressions recorded in situations provoking certain discrete emotions appear more difficult to interpret [27]. Interestingly, evaluator’s experience of dogs does not seem to have a strong influence on the interpretation of a dog’s emotional state when read from the face only [9,27]. However, experience is related to judgements of dogs’ bodily cues [26,28]—perhaps since people with higher dog experience appear better at comprehending dog’s behaviour associated with emotions [29].

Beyond expertise, very little is known about other factors that may affect how human observers evaluate dogs’ facial expressions of emotions. Human perception of conspecific facial expressions, however, is affected by psychological factors such as empathy and personality. Human empathy is generally agreed to comprise emotional empathy, cognitive empathy, and the separation of the self from the other [30]. Emotional empathy is further divided into emotional contagion (originating from the rather automatically triggered sharing of the other’s affective state, likely a phylogenetically old mechanism [31]) and empathic concern (expressing a worry about others’ wellbeing); instead, cognitive empathy involves a theory of mind -like meta-representation of the other’s emotional state [32]. Observer’s empathic abilities affect their evaluation of emotions from human facial expressions: people with higher empathic scores (usually in emotional empathy or total empathy measures) are more accurate in recognizing emotional facial expressions of other humans [33,34]. They also recognize the emotions earlier [35] and estimate the intensity of the emotions higher [36].

Also personality factors have an effect on the perception of human facial expressions. Human personality is characterized by individual differences arising from a person’s socio-cultural development, including person’s attitudes, social relationships and habits [37]. Personality can be divided into five factors, often collectively referred to as the Big Five: these factors are generally stable over time [38–40] and they are equally heritable [41]. Two of the factors, *extraversion* (characterized by persons’ assertiveness, activity, enthusiasm and positive emotions) and *neuroticism* (characterized by tenseness, self-consciousness, emotional vulnerability and moodiness) are often connected to a persons’ perception of the emotional world. People with higher *extraversion* generally evaluate happy human facial expressions as being happier than do those with lower extraversion [42]. In neuroscientific studies, *extraversion* correlates with amygdala activation during observation of positive and *neuroticism* with negative emotional images [43,44], and neuroticism modulates the connectivity between amygdala and prefrontal cortex during processing negative facial expressions [45].

It is not clear how the psychological factors such as personality or empathy may affect emotion evaluation from the expressions of non-human species. However, since dog owners represent the dog emotions with comparable dimensions as human emotions [46], we can expect observer’s own psychological abilities to affect perception of dog facial expressions similarly as expressions of humans. Supporting this view, human empathy appears to generalize toward other species [47], and veterinarian’s empathic abilities affects his/her evaluation of animal pain [48]. Since empathy has also been suggested to affect interpretation of dog behavior [49], it may affect also perception of emotions from dog facial expressions.

In this study, we investigated how humans estimate facial expressions of the non-human species of dogs. The subjects rated the valence, arousal and six basic emotions [50] from images of human and dog faces. The effects of background factors as personality [51], empathy [52], animal-directed empathy [48] and experience of dog behavior were also assessed, and the subject response times were measured.

Our study had two main aims. The first aim was to characterize how human observers detect and rate affect in dog negative, positive and neutral facial expressions in comparison with human expressions and ambiguous non-emotional stimuli, and which discrete emotions are reported in the expressions. The second aim was to clarify the effect of psychological factors (empathy, personality and experience in dog behavior) on the subjects’ ratings. To refine the latter, we were interested in 1) whether the psychological factors affect the intensity of valence and arousal ratings of dog and human facial expressions, 2) whether the psychological factors affect the happiness ratings of Pleasant Dog and Human faces similarly, and 3) whether the psychological factors affect the anger/aggressiveness ratings of Threatening Dog and Human faces similarly.

Based on a previous study [9], we predicted that human subjects evaluate the Pleasant and Threatening facial expressions of dogs similarly to the facial expressions of humans, associating Pleasant expressions mostly with happiness and Threatening expressions mostly with anger/aggressiveness. Furthermore, based on empathy [33–36] and personality [42–45] affecting the perception of human facial expressions, we expected the evaluation of dog facial expressions to be likewise affected by these psychological factors. In particular, we expected the subjects with higher emotional empathy to evaluate happiness higher in Pleasant and anger/aggressiveness in Threatening faces (based on [33–36]); subjects with higher extraversion to evaluate Pleasant faces as happier and subjects with high neuroticism to evaluate Threatening faces as more angry/aggressive (based on [42–45]). Additionally, based on previous studies [9,26,28,53], we expected that dog expertise may have a moderate effect on evaluating dog facial expressions.

## Materials and Methods

### Ethics statement

The study had a prior approval by the ethics committee of Aalto University (approval in the Board Meeting held on the 6th of March, 2014), and investigation was conducted according to the principles expressed in the Declaration of Helsinki. All study participants gave their written informed consent prior to the experiment.

### Subjects

Altogether 34 healthy volunteers participated in the experiment: 15 males and 19 females aged 25–46 years, 37 ± 6 years (mean ± SD). Subjects had no known past or present neurological or mental disorders and they were not compensated monetarily. All but one participant were right-handed according to the Edinburgh Handedness Inventory [54]: on the scale from –1 (left) to +1 (right), the mean ± SD score was 0.85 ± 0.28 (range from –0.60 to 1). In total, 26/34 subjects had had a dog in the family; 16/34 owned or had owned a dog/dogs that they were primarily responsible for; and 15/34 subjects were active in dog-related hobbies (such as agility, obedience training, or game hunting).

### Stimuli

Stimuli comprised altogether 80 different images: 30 color photographs of dog faces (pre-categorized on the basis of the facial expression as Threatening Dogs × 10, Neutral Dogs × 10, Pleasant Dogs × 10); 30 photographs of human faces (likewise pre-categorized as Threatening Humans × 10, Neutral Humans × 10, Pleasant Humans × 10); and as additional controls, 10 images of general household Objects and 10 abstract Pixel images that were phase-scrambled from the neutral dog faces. In all human categories, half of the images represented female and half male actors; both human and dog faces were of unfamiliar adults, and both human and dog images were manipulated to contain only the face, fur around the face, and ears. A between-actor design of stimuli (one actor per image) was employed; altogether, 30 human and 30 dog actors were depicted, 10 in each different emotional category. The dog images represented altogether 24 breeds + 2 mongrels.

Since human empathy and mental state attribution generalizes toward animals [47,55] as well as inanimate categories such as objects, fruits or ambiguous stimuli (e.g [56,57,58]), the inanimate categories of Objects and Pixels served as additional controls. Objects and phase-scrambled images are the most widely utilized controls in the study of human face perception: objects since they provide a category that humans see in their daily life about as often as faces of other people and phase-scrambled images as they provide a well-controlled physiological match to the stimuli (see e.g. [59,60–62]). Both of these inanimate categories function here as an important informant of the generalization effects of personality and empathy.

The human facial images were acquired from royalty-free online sources (e.g. BigStock^TM^, 123RF®), dog facial images from online sources and from photographer Aino Pikkusaari, and object images (e.g. a toaster, a coffee maker, a wall clock, a backpack) from the BOSS database [63]. The face stimuli were characterized with facial action coding system (FACS, [64]) and dog-FACS [8], and they were first used in a study examining dogs’ gazing patterns; for more details of the human and dog faces, see [12].

### Stimulus presentation

Stimuli were displayed on a 14” LCD laptop screen from a normal viewing distance, were approximately 15 x 16 cm^2^ in size, and were overlaid on a gray background. They were presented in a pseudorandomized order (with no more than three subsequent images belonging to the same category), and the stimulus presentation was controlled with Presentation® software (http://nbs.neuro-bs.com/) running a script that recorded both the subject response and the response time. Each image was shown until the subject had responded to all questions (see below), and the next image followed immediately after.

### Subject task: rating facial expressions

Each subject performed the experiment individually in a standard office room. They were informed that they would see different images containing human and dog faces, objects and abstract pixel images. They were instructed to explore the images freely and, for each image, to estimate how the target in the image is feeling or what is the emotional state of the target. The subjects were explained that there were no right answers but that we were interested in their subjective opinions and ratings.

Before the actual experiment, each subject completed a practice session rating four images that were not part of the actual experiment (a dog face, a human face, an object and a pixel image) and confirmed they had understood their task and the procedure. To give the subject full privacy for the task, the experimenter left the room and waited outside during the experiment.

For every image, eight different questions appeared at the bottom of the screen, one at a time, and the subject answered each question by pressing keyboard numeral buttons 1–7. After the subject had pressed a button to answer, the next question appeared on the screen, and after answering all the questions the next image appeared. No time limit was set, thus the subjects could spend however long they wanted at each image/question, but they were encouraged to answer according to their first impressions.

Questions 1 and 2 sampled the valence and arousal of the images (1. Valence rating; 1 = very negative, 7 = very positive; 2. Arousal rating; 1 = not arousing, 7 = highly arousing) and they were always presented first and in the same order. The subsequent questions 3–8 considering the basic, discrete emotional expressions of happiness, sadness, surprise, disgust, fear and anger/aggression (e.g. How much happiness does the image contain? 1 = not at all, 7 = very much) were presented in a randomized order. Generally, subjects answered one question in 3 ± 1 s (mean ± SD). The rationale to rate all the six basic emotions instead of e.g. only happiness and aggressiveness was to obtain a larger variance larger variance in reporting what was perceived, regardless of what is depicted. To illustrate the variety of possible emotions people may attribute to the faces, subjects browsed through the stimulus images, printed on paper, once more at the end of the experiment. They could write in the print images and indicate if they had thought some of the stimuli represented another emotion than the six rated in the experiment. In total, the rating task lasted about 0.5–1 hours, and the experiment in total about 1.5 hours.

### Behavioral questionnaires

After observing and rating the stimuli, the subjects completed several questionnaires: the Big Five Inventory [51] sampling their personality; the Interpersonal Reactivity Index (IRI, [52]) sampling their general human-directed empathy; and animal-directed IRI [48] sampling their animal-directed empathy. The Big Five (BFI) includes five different personality factors: *Extrovertism*, *Neuroticism*, *Agreeableness*, *Openness and Conscientiousness*, of which we focused on the first two due to their pre-established role in emotion detection [65]. Interpersonal Reactivity Index (IRI) samples four factors of empathic abilities: *Perspective-taking* and *Fantasy Scale* sampling cognitive empathy (the ability to cognitively reason the viewpoint of another person without necessarily sharing the emotion), and *Emotional Concern* and *Personal Distress* sampling emotional empathy (sharing the emotion and experiencing concern of another’s wellbeing). On the basis of previous literature [33] and to include both cognitive and emotional factors in the study, we focused on cognitive empathy factor *Perspective-taking* and emotional empathy factor *Emotional Concern* as the main features affecting emotion detection. Finally, the animal-directed IRI comprise the *Perspective-taking* (*ani-PT*) and *Emotional Concern* (*ani-EC*) subscales that were previously re-worded from IRI to concern animals [48]: the first one samples the cognitive perspective-taking abilities (cognitive empathy) of humans toward animals, and the second one samples the affective emotional concern (emotional empathy) of humans toward animals. Both of the subscales were included in our analyses.

Additionally, the subjects answered questionnaires concerning their interest and experience of dogs: *self-rated dog experience* (referred to as *Expertise* measure) in a Visual-Analogue Scale (sampling e.g. the experience in identifying dog behavior; questions 1–4 in S1 Table) as well as *quantified dog exposure* (*Exposure* measure) related to dogs in a 3–5 point multiple choice scale (sampling e.g. ownerships of dogs; questions 1–6 in S2 Table).

### Statistical analysis

Data were analysed with random-intercept Generalized Linear Mixed Models (GLMM) by using Stata® 14 (xtmixed function, www.stata.com; StataCorp LP, Texas, USA). The clustering variable was the subject, taking into account the non-independence of ratings made by the same subject; age and gender were included as fixed effects, and the models were estimated with maximum likelihood method. The variable selection was based on the hypotheses made on the basis of previous literature [9,26,28,33–36,42–45,53].

Subject-rated valence, arousal, and discrete emotions (happiness, sadness, surprise, disgust, fear and anger/aggression) were included as outcome variables, and stimulus categories (Pleasant Dogs, Neutral Dogs, Threatening Dogs, Pleasant Humans, Neutral Humans, Threatening Humans, Objects, Pixels) were included as predictor variables. To obtain the profile of emotions rated in the dog and human facial expressions, the difference between the emotion ratings (happiness, sadness, surprise, disgust, fear, anger/aggressiveness) was calculated for the Pleasant Dog, Neutral Dog, Threatening Dog, Pleasant Human, Neutral Human and Threatening Human expressions. To obtain the comparison of emotions rated for the dog expressions and other stimuli, the difference between the dog expressions (Pleasant Dog, Neutral Dog and Threatening Dog) and other stimulus categories (Pleasant Human, Neutral Human, Threatening Human, Object, Pixel) was also calculated for each of the emotion ratings (happiness, sadness, surprise, disgust, fear, anger/aggressiveness).

Finally, connection of the psychological factors (personality, empathy and expertise) with the subject ratings (arousal, valence, happiness and anger/aggressiveness of the facial expression stimuli) were assessed by including *Extraversion*, *Neuroticism*, *Perspective-taking*, *Empathic concern*, *Animal-directed empathic concern (ani-EC)*, *Animal-directed perspective-taking (ani-PT)*, *Expertise* and *Exposure* as the predictor variables. Similar GLMM model was run for clarifying connections of the psychological factors and the subject response time in rating stimulus valence; since the question of valence was always presented first, we used that as a proxy of the reaction time.

All the results were subjected for corrections of multiple corrections with the false discovery rate (FDR) method [66]; according to the specific hypotheses laid down according the previous literature not using this method (e.g. [33,36]) also the original results are given. The original data can be found in S1 Dataset.

## Results

### Differences of valence and arousal ratings between stimulus categories

#### Valence

**Fig 1:**
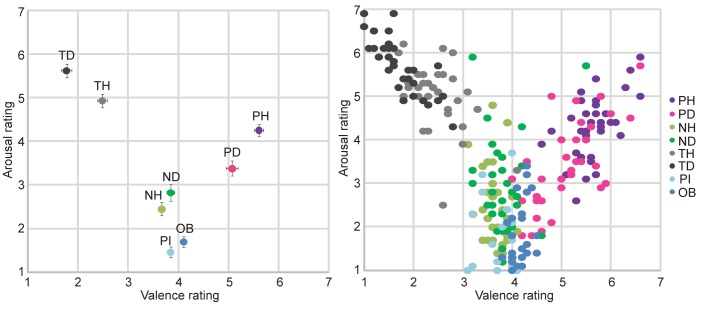
Valence and arousal ratings. Positioning of the subject ratings of human, dog, object and pixel stimuli on the classical valence (x-axis; 1 = negative, 7 = positive) and arousal (y-axis; 1 = low arousal, 7 = high arousal) dimensions. Left: Subject grand mean ratings with SEM, Right: Individual subject mean ratings of each category. PH = Pleasant Humans, PD = Pleasant Dogs, NH = Neutral Humans, ND = Neutral Dogs, TH = Threatening Humans, TD = Threatening Dogs, PI = Pixels, OB = Objects.

Fig 1 illustrates that generally human faces were rated as more positive than dog faces except with neutral expressions, where dog faces were rated as more positive (Fig 1). All results given for valence and arousal survived the correction for multiple comparisons.

Between the dog categories, Pleasant Dogs were rated as more positive than Neutral or Threatening Dogs (β = 1.22, *p* < 0.000 and β = 3.30, *p* < 0.000, respectively), and Neutral Dogs as more positive than Threatening Dogs (β = 2.08, *p* < 0.000). Between dog *vs*. human faces, Pleasant Humans were seen as more positive than Pleasant Dogs (β = 0.54, *p* < 0.000), Neutral Dogs more positive than Neutral Humans (β = 0.18, *p* < 0.05), and Threatening Dogs more negative than Threatening Humans (β = 0.71, *p* < 0.000).

As Fig 1 shows, the valence of Objects and Pixels were rated very similarly than Neutral Dogs and Humans (Fig 1). Objects were seen as more negative than Pleasant Dogs and more positive than Neutral or Threatening Dogs (β = 0.96, *p* < 0.000; β = 0.26, *p* < 0.01 and β = 2.34, *p* < 0.000, respectively). Pixels were rated as more negative than Pleasant and more positive than Threatening Dogs (β = 1.22, *p* < 0.000 and β = 2.07, *p* < 0.000, respectively), but the valence of Pixels did not differ from Neutral Dogs (β = 0.00; *p* = 0.970).

#### Arousal

Fig 1 shows that Threatening faces of both species elicited highest arousal, followed by pleasant and neutral faces; arousal elicited by Pixels or Objects was close to none (Fig 1). Between dog categories, Threatening Dogs were seen as more arousing than Pleasant or Neutral Dogs (β = 2.24, *p* < 0.000; β = 2.80, *p* < 0.000, respectively), and Pleasant Dogs more arousing than Neutral Dogs (β = 0.56; *p* < 0.000). Between dog *vs*. human faces, Pleasant Humans were rated as more arousing than Pleasant Dogs (β = 0.87, *p* < 0.000), Neutral Dogs more arousing than Neutral Humans (β = 0.37, *p* < 0.000), and Threatening Dogs as more arousing than Threatening Humans (β = 0.69; *p* < 0.000).

All dog categories were rated as more arousing than Objects or Pixels (For all comparisons, β > 1.13, *p* < 0.001).

### Range of discrete emotions seen within dog facial expressions and between dog vs. other categories

**Fig 2:**
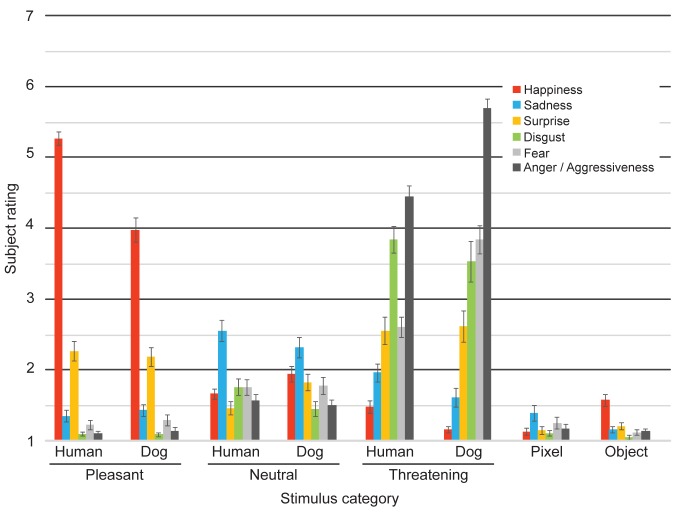
Discrete emotion ratings. Subject ratings of happiness, sadness, surprise, disgust, fear and anger / aggressiveness (on a scale from 1 (min) to 7 (max) for all the stimulus categories (Pleasant Humans, Pleasant Dogs, Neutral Humans, Neutral Dogs, Threatening Humans, Threatening Dogs, Pixel, and Object (Mean ± SEM)).

Ratings of discrete emotions on the facial expressions of dogs followed similar patterns than ratings of humans’ equivalent facial expressions, but the ratings of Pixels and Objects were clearly different from these (Fig 2).

For Pleasant Dogs, happiness was rated higher than the other discrete emotions, whereas disgust and anger/aggression was rated lower than the others (Table 1). Vice versa, for Threatening Dogs, anger/aggression was rated higher than the other emotions, and happiness was rated lowest. By contrast, Neutral Dog faces were more uniformly scored and received higher scores on sadness than other emotions. Ratings of human facial expressions were very similar; Pleasant Humans were rated highest on happiness, Threatening Humans on anger/aggressiveness and Neutral Humans on sadness (all regressions of discrete emotions in human expressions are available in S3 Table).

**Table 1:** Differences of emotion ratings within dog expressions. Planned comparisons of the subject ratings of discrete emotions (Emotion 1 *vs*. Emotion 2) for each of the dog expression categories. Significant differences are marked on the beta values with asterisks (*p < 0.05, **p < 0.01, ***p < 0.001) and the results with p-values above the FDR threshold are written in **bold** type.

		Stimulus		
Emotion 1	Emotion 2	Pleasant Dogs	Neutral Dogs	Threatening Dogs
Happiness	Sadness	2.55***	–0.38*	–0.45***
Happiness	Surprise	1.79***	0.12	–1.45***
Happiness	Disgust	2.89***	0.49**	–2.38***
Happiness	Fear	2.68***	0.17	–2.69***
Happiness	Anger/Aggression	2.84***	0.44***	–4.54***
Sadness	Surprise	–0.76***	0.49**	–1.00***
Sadness	Disgust	0.34***	0.87***	–1.92***
Sadness	Fear	0.14	0.54***	–2.23***
Sadness	Anger/Aggression	0.29***	0.82***	–4.09***
Surprise	Disgust	1.10***	0.37**	–0.92***
Surprise	Fear	0.89***	0.05	–1.23***
Surprise	Anger/Aggression	1.05***	0.32**	–3.09***
Disgust	Fear	–0.21**	–0.32**	–0.31
Disgust	Anger/Aggression	–0.05	–0.05	–2.16***
Fear	Anger/Aggression	0.16**	0.27**	–1.85***

In the free elaboration, where subjects could indicate another emotion than the above six, the following descriptions for individual dog stimuli were given: Pleasant Dogs: excited, satisfied, playful, observant; Neutral Dogs: tense, attentive, alert, stressed, determined, shy, doubtful, tired, humble, bored, disappointed, thoughtful, concentrated, reserved, submissive, embarrassed, content, observant; Threatening Dogs: grimacing, mischief, yawning, defensive.

Pleasant Dogs were rated higher on happiness than Neutral or Threatening Dogs, but Pleasant Humans were rated even happier than Pleasant Dogs (Table 2). Sadness was rated higher in Neutral Dogs *vs*. Pleasant or Threatening Dogs, but Neutral Humans were rated higher in sadness than Neutral Dogs. Surprise, disgust, fear and anger were rated higher in Threatening *vs*. Pleasant or Neutral Dogs, and fear and anger were also rated higher in Threatening Dogs than Threatening Humans.

**Table 2:** Difference of emotion ratings between stimuli. Planned comparisons of the subject ratings of discrete emotions between dog expressions and other stimulus categories (Stimulus 1 *vs*. Stimulus 2). Ratings were compared between dog expressions, dog *vs*. human expressions, and dog expressions *vs*. objects and pixels. Significant differences are marked on the beta values with asterisks (*p < 0.05, **p < 0.01, ***p < 0.001) and the results with p-values above the FDR threshold are written in **bold** type.

		Emotion					
Stimulus 1	Stimulus 2	Happiness	Sadness	Surprise	Disgust	Fear	Anger/Aggression
Pleasant Dogs	Neutral Dogs	2.04***	–0.89***	0.36***	–0.36***	–0.48***	–0.36***
Pleasant Dogs	Threatening Dogs	2.82***	–0.18	–0.43*	–2.45***	–2.55***	–4.56***
Neutral Dogs	Threatening Dogs	0.78***	0.71***	–0.79***	–2.09***	–2.07***	–4.20***
Pleasant Dogs	Pleasant Humans	–1.29***	0.08	–0.08	–0.01	0.07	0.03
Neutral Dogs	Neutral Humans	0.28**	–0.24	0.36***	–0.31***	0.02	–0.06
Threatening Dogs	Threatening Humans	–0.31***	–0.35**	0.06	–0.31	1.24***	1.25***
Pleasant Dogs	Objects	2.40***	0.27**	0.98***	0.04	0.18*	0.00
Neutral Dogs	Objects	0.37**	1.16***	0.62***	0.40***	0.65***	0.36***
Threatening Dogs	Objects	–0.41***	0.46***	1.41***	2.49***	2.73***	4.56***
Pleasant Dogs	Pixels	2.85***	0.04	1.04***	–0.02	0.04	–0.04
Neutral Dogs	Pixels	0.81***	0.93***	0.68***	0.34***	0.52***	0.33***
Threatening Dogs	Pixels	0.03	0.22	1.46***	2.43***	2.59***	4.52***

### Psychological factors affecting the responses for stimulus arousal, valence, happiness and anger/aggressiveness

The emotional empathy factor *Emotional Concern* (EC) covaried negatively with the valence ratings of Threatening Dogs, Threatening Humans, and Pleasant Humans, and positively with the arousal ratings of Threatening Dogs (see all regression coefficients in Table 3). Furthermore, *Emotional Concern* was positively associated with anger/aggressiveness ratings of Threatening Humans and Threatening Dogs, as well as with happiness ratings of Pleasant Humans. Animal-directed *Emotional Concern* (aniEC) covaried similarly with anger/aggressiveness of Threatening Dogs, but not with other factors.

**Table 3:** Connections of psychological factors to the stimulus ratings. Ratings of arousal, valence, happiness and anger/aggressiveness were predicted with Extraversion (Ex), Neuroticism (Neur), Emotional Concern (EC), Perspective-taking (PT), animal-directed Emotional Concern (aniEC) and animal-directed Perspective-taking (aniPT) factors. Connections of dog stimulus ratings with dog experience were obtained with dog expertise (Expe) and dog exposure (Expo) predictors. Significant regression coefficients (beta values) are marked with asterisks (*p < 0.05, **p < 0.01, ***p < 0.001) and the results with p-values above the FDR threshold are written in **bold** type.

		Empathy		Animal empathy		Personality		Dog expertise	
Rating	Stimulus	EC	PT	aniEC	aniPT	Ex	Neur	Expe	Expo
Valence	Pleasant Humans	0.04*	0.01	0.01	0.00	0.05	0.00		
	Pleasant Dogs	0.03	0.00	0.02	0.00	0.13	0.23	0.00	0.03
	Neutral Humans	–0.01	–0.01	0.00	0.01	0.00	0.07		
	Neutral Dogs	0.02	–0.01	0.01	0.02	0.18	0.11	0.00	0.03*
	Threatening Humans	–0.07**	–0.06*	–0.02	0.02	0.04	0.08		
	Threatening Dogs	–0.05**	–0.01	–0.02	0.02	0.09	–0.16	0.00	–0.01
	Object	–0.01	–0.01	–0.01	–0.01	–0.03	0.07		
	Pixel	–0.01	–0.01	0.00	–0.02	–0.10	0.01		
Arousal	Pleasant Humans	0.04	–0.01	–0.01	–0.02	0.25	–0.40		
	Pleasant Dogs	0.01	–0.06	0.03	0.01	0.39	–0.07	0.00*	0.03
	Neutral Humans	–0.01	–0.02	0.00	0.02	0.41	–0.53***		
	Neutral Dogs	–0.01	–0.06	0.02	0.03	0.53	–0.52*	0.00	0.02
	Threatening Humans	0.08	0.04	0.01	–0.06	0.07	–0.36		
	Threatening Dogs	0.07***	–0.02	0.02	–0.04	–0.10	0.13	0.00	0.02
	Object	–0.04	–0.07*	–0.03	0.01	0.20	–0.03		
	Pixel	–0.01	–0.05*	–0.01	0.03	0.24	0.05		
Happiness	Pleasant Humans	0.09***	0.08**	0.02	0.00	0.10	–0.34*		
	Pleasant Dogs	0.07	0.05	0.04	0.07	0.36	–0.25	0.00	0.04
	Neutral Humans	0.01	0.03	0.01	0.00	–0.05	–0.20*		
	Neutral Dogs	0.03	0.00	0.02	0.04	0.30	–0.17	0.00*	0.03
Anger/ Aggressiveness	Threatening Humans	0.12***	0.12***	0.02	–0.01	–0.08	–0.30		
	Threatening Dogs	0.08**	0.06	0.04*	0.01	–0.11	0.05	0.00	0.02
	Neutral Humans	0.02	0.01	0.02	0.03	0.24*	–0.24		
	Neutral Dogs	0.02	0.00	0.02	0.02	0.10	–0.23*	0.00	0.01

The cognitive empathy factor *Perspective-taking* had no connections to ratings of dog expressions (Table 3). It covaried negatively with valence of Threatening Humans and the arousal ratings of the inanimate Object and Pixel categories. *Perspective-taking* also covaried positively with the happiness ratings of Pleasant Humans as well as anger/aggressiveness ratings of Threatening Humans. Animal-directed *Perspective-taking* (*ani-PT)* had no connection to the ratings.

The personality factor *extraversion* covaried positively with the anger/aggressiveness ratings of Neutral Humans, and *neuroticism* had a negative connection with a number of ratings: the arousal ratings of Neutral Humans and Dogs; the happiness ratings of Pleasant and Neutral Humans; and the anger/aggressiveness ratings of Neutral Humans and Dogs.

*Self-rated dog experience* (*Expertise*) covaried positively with the arousal ratings of Pleasant Dogs and happiness ratings of Neutral Dogs, and the *quantified dog exposure* (*Exposure*) covaried with the valence ratings of Neutral Dogs.

As side findings, minor connections (with no specific hypotheses and surviving no corrections for multiple comparisons) were found with the ratings of valence and arousal and subject age or gender. Older subjects rated Happy Humans and Dogs as higher in arousal (β = 0.38, *p* < 0.05; β = 0.48, *p* < 0.01, respectively); Neutral Dogs as more positive in valence, and Pixels more negative in valence (β = 0.35, *p* < 0.05; β = –0.35, *p* < 0.05) than younger subjects. Female subjects rated Aggressive Dogs and Pixels more negative than males (β = –0.36, *p* < 0.05; β = –0.37, *p* < 0.05). However, none of these results survived the multiple comparison correction with FDR, and we had no specific hypotheses about them, thus they are reported here as additional information only.

Additionally, we assessed the connections of subject response times to valence ratings (which were always asked first for each image). The response times covaried negatively with *Emotional Concern* factor in rating Pleasant Humans, Neutral Dogs, Threatening Humans and Threatening Dogs, thus the responses for these were quicker in subjects with high emotional empathy than those with lower emotional empathy (Table 4). *Animal-directed Emotional Concern* showed a similar negative covariation, but only with rating Threatening Humans.

**Table 4:** Connections of psychological factors to the response times in rating stimulus valence. Response times for valence estimation were tested with Emotional Concern (EC), Perspective-taking (PT), animal-directed Emotional Concern (aniEC) and animal-directed Perspective-taking (aniPT), Extraversion (Ex) and Neuroticism (Neur). Connections of dog stimuli with dog experience were obtained with dog expertise (Expe) and dog exposure (Expo) measures. Significant regression coefficients (beta values) are marked with asterisks (*p < 0.05, **p < 0.01, ***p < 0.001).

	Empathy		Animal empathy		Personality		Dog expertise	
Stimulus	EC	PT	aniEC	aniPT	Ex	Neur	Expe	Expo
Pleasant Humans	-1336**	-1043	-941	1317	8711	-3664		
Pleasant Dogs	-451	114	-621	1275	8974	-6917	3	70
Neutral Humans	-1139	-847	-980	1123	8492	-9091		
Neutral Dogs	-1934**	-949	-704	1545	4839	-4728	26	562
Threatening Humans	-2313**	-1441	-1226*	1041	8372	-4432		
Threatening Dogs	-2423**	-734	-1096	1851	9579	-6581	23	704
Object	-1863	-1428	-1569	876	5412	110		
Pixel	-734	-886	-533	2340	13730	-6791		

## Discussion

### Human recognition of emotions from dog faces

The ratings of both species followed a very similar pattern, suggesting the same face perception [67–69] and affective mechanisms [70] to be engaged in the evaluation. As expected, the Pleasant faces of both humans and dogs were rated as positive with moderate arousal; Threatening faces were rated as negative with high arousal, and the inanimate Object and Pixel categories were rated as neither positive nor negative, with very little arousal. In addition to the valence and arousal ratings, we collected the subjects’ ratings of the basic discrete emotions seen in the facial expressions to gather a variability of how people generally perceive the expressions. Although the subjects had a range of possibilities, our data confirm that the subjects observed the Pleasant Dog and Human faces as happy, Neutral Dog and Human faces not high in any emotion but as slightly sad, and Threatening Dog and Human faces as angry/aggressive. The ratings are in line with the well-known literature on human faces (e.g. [71]), and they also agree with a previous study by Schirmer and colleagues [9], who showed that positive and negative expressions of dogs are discriminated as accurately as those of human children.

We also observed a conspecific bias in Pleasant, and a non-conspecific bias in Threatening expressions: Pleasant Humans were rated happier than Pleasant Dogs and Threatening Dogs more aggressive than Threatening Humans. Since we do not have the subjective emotion ratings from the model dogs and humans within the stimuli, we cannot reflect on the full accuracy of these estimates. However, the results are ecologically meaningful, demonstrating the more positive value of the conspecifics, and generally reflecting a threat from non-conspecifics as of high concern. Our recent eye-tracking results on dogs with the same set of stimuli also showed a species-specific bias: dogs tended to avoid the Threatening Human faces [12]. We interpreted the result as likely resulting from the appeasing behavior of dogs, but the current human data suggests a more general mechanism in perceiving non-conspecific threat may also play a part. Alternatively, Threatening Dogs were rated here as more arousing than Threatening Humans, which may affect the species-specific bias in both humans and dogs. Investigating the effect further in the future would benefit from more precise brain imaging methods.

Although Threatening Dog faces were rated highest on aggression, they were also estimated as somewhat fearful and disgusted. Since the situation in which our stimuli were photographed is unknown and dog aggression is often exhibited in fearful situations [72], it is likely that some of the images of Threatening Dogs capture also fear. However, disgust provoked by a disliked taste represents a rather distinct emotion from aggression. Previously, a dog facial expressions filmed in a situation likely to provoke disgust (obtaining a medicine) received higher ratings of both anger and sadness than disgust from the human observers [27]. Additionally, aggression appears to be badly recognized from videos of dog behavior by humans, and confused with other emotions [24]. The expression of disgust appears differently recognizable from other basic emotions in dogs, since only 34–54% of dog owners report having seen the expression of disgust in dogs, whereas other basic emotions are reported by 65%–100% of dog owners [15,16]. Together, these findings propose that disgust presented by a dog facial expression is quite difficult for humans to detect, and it is easily confused with aggression.

### Influence of psychological factors on emotion evaluation

Our results show that the perception of emotion from dog faces—as well as from human faces—is a multifaceted phenomenon, influenced by psychological factors of the observers. We found that subjects’ empathy, personality and experience affected the estimation of emotions. Emotional empathy (*emotional concern*, EC) had the clearest influence on estimations, covarying both with Threatening facial expressions of both species and Pleasant human facial expressions, but importantly, never with the Neutral faces nor the inanimate Objects or Pixels. Subjects with higher EC also made these valence judgments spontaneously quicker, although it should be noted that we measured response time instead of reaction time. Animal-directed *emotional concern* (aniEC) mimicked the connection of general EC regarding anger/aggressiveness ratings of Threatening Dogs, and it similarly lead to quicker responses in evaluating Threatening Humans. Recently, emotional empathy has been found to enhance the accuracy [33,34] and speed [73] of recognizing human emotional expressions. In addition, the ratings of emotion intensity also correlate positively with the emotional empathy of the rater [36]. Our results strongly agree with the previous literature, broadening the effect of subject’s emotional empathy also to estimation of emotion from dog facial expressions.

Many of the previous studies have only explored the effect of emotional empathy on emotion estimation, but we also studied the possible effect of cognitive empathy. The subjects’ cognitive empathy (*perspective-taking*) correlated positively with the estimated happiness of Pleasant Humans and anger of Threatening Humans, and negatively with the valence of Threatening Humans, but had no connection to the ratings of dog facial expressions. In agreement with this, a previous study found a connection between cognitive empathy and the accuracy of estimating human emotions [74]. Interestingly, we also found that the higher was the cognitive empathy of the subject, the less arousal she/he estimated in the inanimate Object and Pixel categories. A previous study has suggested that general empathy affects evaluation of abstract visual stimuli [58], but our data do not support this. Instead, our results suggest that cognitive empathy can fine-tune the estimation of emotions from human faces, but cognitive empathy also has a “reality check” effect on considering inanimate objects’ emotions, providing one with the ability to separate one’s own emotions from those of another. Importantly, cognitive empathy was not connected to evaluating emotional states of dogs, suggesting either that emotional empathy is more functional when considering the inner states of non-humans, or that cognitive perspective-taking is ill-equipped for considering the perspective of a living creature with a likely different mental construct.

Previously, personality factor *extraversion* has been connected to amygdala activation during observation of positive images and happy human faces and *neuroticism* during observation of negative images [43,44]. In our data, we found personality effects on estimating emotions from the Pleasant and Neutral face categories: the higher were the *extraversion* scores of the subject, the higher they rated the anger/aggressiveness of Neutral Humans, and the higher the *neuroticism* scores, the smaller they rated the arousal and anger/aggressiveness of Neutral Human and Dog faces, as well as happiness of Pleasant and Neutral Humans. Although the *extraversion* connection to higher anger/aggressiveness ratings was unexpected considering the previous findings of its connections to higher ratings of happy faces [42], the results were more plausible when put together with the *neuroticism* connections. Here, the enthusiasm of the *extraversion* and suspiciousness of the *neuroticism* appeared to consider the subject task much more than the actual stimuli. In other words, the subjects higher in *neuroticism* tended to ponder the questions regarding the ambiguous Neutral faces rather critically (“It is probably meant to be happy but I don’t think it is so happy” or “It is probably meant to be angry but I don’t think it is so angry”), whereas the subjects higher in *extraversion* did not take it so seriously (“Anger? Well, maybe it is somewhat angry”). Thus, the findings are in line with the connection of *extraversion* and *neuroticism* to the view of the world: the former had positive and the latter negative application of the task to the ratings of the faces.

### Does dog expertise affect judging emotion from dog facial expressions?

In addition to the more basic psychological factors, we were also interested in how the attitudes, experience and education in dog behaviour affect the estimation of dog emotional expressions. Our subjects having more experience or exposure on dogs estimated the Neutral Dogs as more positive and happier, and the Pleasant Dogs as having higher arousal than did the less experienced subjects, but experience did not affect judging aggressiveness of Threatening Dogs. In line with this, previous studies also suggest that experience on dogs is not necessary for being able to detect basic facial expressions of dogs [9,27]. However, experienced subjects have rated aggressive dog faces as less aggressive previously [27], which perhaps taps the same phenomenon as witnessed in our study: people experienced with dogs generally have a more positive attitude towards dogs, which surfaces when estimating emotions from dog facial images. Alternatively, the expressions of positive emotions in dogs may be subtle and especially positive and relaxed states of dogs are difficult for dog owners to describe [22], thus people with higher experience with dogs may better recognize relaxed, non-threatening expressions.

Although experience and knowledge appear to have little effect on detection of dogs’ basic facial expressions, they affect the interpretation of dog behaviour in more complex situations involving the whole animal and probably with the combination of different bodily gestures. In our previous study, people experienced with dogs had similar brain activations to observing interacting dogs and interacting humans, whereas in lay people, the activation was specific to interacting humans [28]. Highly experienced dog trainers can also judge the fear of dogs from whole-body videos more accurately than lay people or even dog owners [26]. Recent studies also suggest that a combination of dog expertise, empathy and general sensitivity for nonverbal behaviour may affect human interpretation of dog behaviour [49,75].

### Biological similarity, anthropomorphism or experience?

The current data shows that the human evaluations of dog and human emotional expressions are influenced by the evaluator’s psychological factors, but the accuracy of the emotion estimation is a more complex question regarding emotional expressions of non-humans, and beyond the scope of the current study. When studying humans, persons photographed for the stimuli can be asked to rate their emotions in the same scale than used later by the observers, and match these two answers. However with animals, this is not possible, and it is difficult to label the animal's exact subjective emotion and estimate its intensity with certainty. Furthermore, we should be also aware of the human social cognition machinery that affects our perception of living creatures as well as constructs with artificial intentionality (see e.g. [76,77]). Thus, in studying animal emotions, it is important to ask: Where lies the border between comprehending the actual biological similarities of other species and merely extending human-centered anthropomorphism, provoked by the human social cognition machinery, to non-human species?

Reading emotions from the faces of non-conspecifics are likely to be based on detecting the gestural cues of facial muscles of the other—probably in the same manner as from conspecifics humans, and powered by the same underlying cognitive machinery including face perception [67–69] and affective responding [70]. Likewise, humans utilize similar cues in assessing valence from both dog and human vocalizations [78]. However, the dimensions of faces differ between the species and the function of facial muscles of non-conspecifics may not match their equivalent function in the human face, thus giving space for anthropomorphic interpretations. In these cases, one would expect the knowledge of dog behavior to affect the judgments, since people tend to anthropomorphize more in situations where they have very little educational knowledge of the actor, and/or they have a high need to establish social connections [79].

Indeed, lay people tend to give more anthropomorphic descriptions of dog behavior than do dog-experienced people [80,81] although the effect is not always clear [24]. Nevertheless, according to the previous and our results, reading the basic facial expressions of dogs are generally agreed on with dog experts and lay people with only minor deviations, suggesting that anthropomorphic interpretations are maybe more prominent when considering secondary emotions or the body expression of a whole animal instead of just the face.

In conclusion, our current research shows that human subjects classify dog facial expressions of the pleasant-threatening axis much in the same manner than human facial expressions, and more importantly, the estimation of emotions in both species are affected mainly by the same psychological variables. Emotional empathy affects both the speed and intensity of rating dogs’ emotional facial expressions, and experience with dogs biases positively evaluation of dogs’ non-threatening emotional expressions.

## Supporting Information

S1 Table Dog expertise. Self-rated dog expertise questionnaire, answered as a visual-analogue scale from 0 to 100.
(DOCX)

S2 Table Dog exposure and hobbies. Questionnaire answered as a 3–5 point closed-end multiple choice scale.
(DOCX)

S3 Table Discrete emotions rated within human expressions. Planned comparisons of the subject ratings of discreet emotions in different human expressions. Significant differences are marked on the beta values with asterisks (*p < 0.05, **p < 0.01, ***p < 0.001).
(DOCX)

S1 Dataset The supporting data including empathy, personality and expertise scores and emotion ratings. (XLSX)
