# Supplementary material for: Human Empathy, Personality and Experience Affect the Emotion Ratings of Dog and Human Facial Expressions
Source: PLoS One. 2017 Jan 23;12(1):e0170730. doi: 10.1371/journal.pone.0170730 (PMC5257001; doi:10.1371/journal.pone.0170730)
Supplement: S1 Table — Self-rated dog expertise questionnaire, answered as a visual-analogue scale from 0 to 100. (DOCX) [file pone.0170730.s001.docx]

### Supplementary Table S1.

**Dog expertise.** Self-rated dog expertise questionnaire, answered as a visual-analogue scale from 0 to 100.

| Nr | Question | Sample |
| --- | --- | --- |
| 1 | How much do you like dogs? | emotional interest |
| 2 | How much are you interested about dogs? | cognitive interest |
| 3 | How much are you oriented towards dog behavior? (training, reading, discussing) | cognitive self-training |
| 4 | How experienced are you in identifying dog behavior? | practical self-training |
| 5 | How much are you interested about animal behavior in general? | general cognitive interest |
| 6 | How much are you interested about human behavior in general? | human-directed cognitive interest |
