# Supplementary material for: Human Empathy, Personality and Experience Affect the Emotion Ratings of Dog and Human Facial Expressions
Source: PLoS One. 2017 Jan 23;12(1):e0170730. doi: 10.1371/journal.pone.0170730 (PMC5257001; doi:10.1371/journal.pone.0170730)
Supplement: S2 Table — Questionnaire answered as a 3–5 point closed-end multiple choice scale. (DOCX) [file pone.0170730.s002.docx]

### Supplementary Table S2.

**Dog exposure and hobbies.** Questionnaire answered as a 3–5 point closed-end multiple choice scale.

| Nr | Question | Multiple choices | Sample |
| --- | --- | --- | --- |
| 1 | Have you had a dog in your family? If yes, for how many years have you had a dog/dogs in your family? | 1. Not at all  2. Under 1 years  3. 1–5 years  4. 6–10 years  5. Over 10 years | childhood dog,  dog in the family |
| 2 | Have you been responsible for a dog in your family? If yes, for how many years have you been responsible for a dog/dogs in your family? | 1. Not at all  2. Under 1 years  3. 1–5 years  4. 6–10 years  5. Over 10 years | responsibility for a dog |
| 3 | How many dogs have you had (in your family) altogether? | 1. Not at all  2. 1  3. 2–4  4. 5 or more | lifelong dog interest/exposure |
| 4 | How many dogs have you had (in your family) concurrently? | 1. Not at all  2. 1  3. 2 or more | exposure to dog-to-dog interaction |
| 5 | Have you had hobbies related to dogs? If yes, for how long altogether? | 1. Not at all  2. Under 1 years  3. 1–5 years  4. 6–10 years  5. Over 10 years | interest/exposure to dog behavior |
