# Supplementary material for: Human Empathy, Personality and Experience Affect the Emotion Ratings of Dog and Human Facial Expressions
Source: PLoS One. 2017 Jan 23;12(1):e0170730. doi: 10.1371/journal.pone.0170730 (PMC5257001; doi:10.1371/journal.pone.0170730)
Supplement: S3 Table — Planned comparisons of the subject ratings of discreet emotions in different human expressions. Significant differences are marked on the beta values with asterisks (*p < 0.05, **p < 0.01, ***p < 0.001). (DOCX) [file pone.0170730.s003.docx]

### Supplementary Table S3.

**Differences of emotion ratings within human expressions.** Comparisons of the subject ratings of discrete emotions between different human expressions. Significant differences are marked on the *beta values* with asterisks (**p* < 0.05, ***p* < 0.01, ****p* < 0.001) and the results with p-values above the FDR threshold are written in **bold** type.

|  |  | Stimulus | | |
| --- | --- | --- | --- | --- |
| Emotion 1 | Emotion 2 | Pleasant Humans | Neutral Humans | Threatening Humans |
| Happiness | Sadness | **3.92***** | **–0.89***** | **–0.49***** |
| Happiness | Surprise | **3.00***** | 0.21 | **–1.08***** |
| Happiness | Disgust | **4.17***** | –0.09 | **–2.37***** |
| Happiness | Fear | **4.04***** | –0.09 | **–1.14***** |
| Happiness | Anger/Aggression | **4.16***** | 0.10 | **–2.97***** |
| Sadness | Surprise | **–0.91***** | **1.09***** | **–0.59***** |
| Sadness | Disgust | **0.25***** | **0.79***** | **–1.88***** |
| Sadness | Fear | 0.13** | **0.80***** | **–0.65***** |
| Sadness | Anger/Aggression | **0.24***** | **0.99***** | **–2.49***** |
| Surprise | Disgust | **1.17***** | –0.30** | **–1.29***** |
| Surprise | Fear | **1.04***** | –0.29** | –0.06 |
| Surprise | Anger/Aggression | **1.16***** | –0.11 | **–1.89***** |
| Disgust | Fear | –0.13** | 0.01 | **1.23***** |
| Disgust | Anger/Aggression | –0.01 | 0.19** | **–0.60***** |
| Fear | Anger/Aggression | 0.12** | 0.19** | **–1.84***** |

In the free elaboration, where subjects could indicate another emotion than the above six, the following descriptions for individual human stimuli were given: Pleasant Humans: shy, glee/Schadenfreude, posing, uncertain, confused; Neutral Humans: absent-minded, thoughtful, tired, proud, contempt, pondering, despise, painful, judgmental; Threatening Humans: anguish, glee/Schadenfreude, teasing, devilish, rock-aggression, clowning, painful, acting, emanation.
